# Using functional principal component analysis (FPCA) to quantify sitting patterns derived from wearable sensors

**DOI:** 10.1186/s12966-024-01585-8

**Published:** 2024-04-26

**Authors:** Rong W. Zablocki, Sheri J. Hartman, Chongzhi Di, Jingjing Zou, Jordan A. Carlson, Paul R. Hibbing, Dori E. Rosenberg, Mikael Anne Greenwood-Hickman, Lindsay Dillon, Andrea Z. LaCroix, Loki Natarajan

**Affiliations:** 1 Herbert Wertheim School of Public Health and Human Longevity Science, University of California at San Diego, 9500 Gilman Drive, La Jolla, 92093 California USA; 2 Division of Public Health Sciences, Fred Hutchinson Cancer Research Center, 1100 Fairview Ave N, Seattle, 98109 Washington USA; 3 Center for Children’s Healthy Lifestyles and Nutrition, Children’s Mercy Kansas City, 610 E. 22nd St., Kansas City, 64108 Missouri USA; 4 Department of Kinesiology and Nutrition, University of Illinois Chicago, 1919 W Taylor St, Chicago, IL 60612 USA; 5 Kaiser Permanente Washington Health Research Institute, 1730 Minor Ave, Suite 1600, Seattle, 98101 Washington USA

**Keywords:** Functional Principal Component Analysis (FPCA), Multilevel FPCA, Sedentary Behavior (SB), Accelerometer

## Abstract

**Background:**

Sedentary behavior (SB) is a recognized risk factor for many chronic diseases. ActiGraph and activPAL are two commonly used wearable accelerometers in SB research. The former measures body movement and the latter measures body posture. The goal of the current study is to quantify the pattern and variation of movement (by ActiGraph activity counts) during activPAL-identified sitting events, and examine associations between patterns and health-related outcomes, such as systolic and diastolic blood pressure (SBP and DBP).

**Methods:**

The current study included 314 overweight postmenopausal women, who were instructed to wear an activPAL (at thigh) and ActiGraph (at waist) simultaneously for 24 hours a day for a week under free-living conditions. ActiGraph and activPAL data were processed to obtain minute-level time-series outputs. Multilevel functional principal component analysis (MFPCA) was applied to minute-level ActiGraph activity counts within activPAL-identified sitting bouts to investigate variation in movement while sitting across subjects and days. The multilevel approach accounted for the nesting of days within subjects.

**Results:**

At least 90% of the overall variation of activity counts was explained by two subject-level principal components (PC) and six day-level PCs, hence dramatically reducing the dimensions from the original minute-level scale. The first subject-level PC captured patterns of fluctuation in movement during sitting, whereas the second subject-level PC delineated variation in movement during different lengths of sitting bouts: shorter (< 30 minutes), medium (30 -39 minutes) or longer (> 39 minute). The first subject-level PC scores showed positive association with DBP (standardized $\hat{\beta }$: 2.041, standard error: 0.607, adjusted *p* = 0.007), which implied that lower activity counts (during sitting) were associated with higher DBP.

**Conclusion:**

In this work we implemented MFPCA to identify variation in movement patterns during sitting bouts, and showed that these patterns were associated with cardiovascular health. Unlike existing methods, MFPCA does not require pre-specified cut-points to define activity intensity, and thus offers a novel powerful statistical tool to elucidate variation in SB patterns and health.

**Trial registration:**

ClinicalTrials.gov NCT03473145; Registered 22 March 2018; https://clinicaltrials.gov/ct2/show/NCT03473145; International Registered Report Identifier (IRRID): DERR1-10.2196/28684

**Supplementary Information:**

The online version contains supplementary material available at 10.1186/s12966-024-01585-8.

## Background

Studies across the spectrum of public health and biomedical research have linked sedentary behavior (SB) to poor health [1–3]. Interventions and strategies have been developed to reduce SB in an effort to improve health benefits within the population [1, 4, 5]. To measure SB reliably, accurately and cost-effectively, it is well established that sensor based accelerometers are the method of choice [6–8]. However, there are a plethora of such devices that are each used individually to measure SB. SB is defined as energy expenditure $\le$ 1.5 metabolic equivalents (low movement) and a seated, reclined, or lying position (posture) during waking hours [9, 10]. Devices used to measure SB differ in the information that they capture (e.g., posture versus energy expenditure), and hence are not always concordant. Combining SB measures across devices could lead to more accurate SB assessment and also provide additional insights into SB patterns. The latter is the focus of this work.

We briefly review two popular devices for SB measurement in health behavior studies. ActiGraph GT3X+ (ActiGraph LCC, Pensacola, FL, USA) is a commonly used hip- or wrist-worn research-grade wearable accelerometer to measure movement based on acceleration across vertical, horizontal, and perpendicular axes [6, 11]. These accelerations are usually sampled at fine granularity (e.g., 10Hz, or 30Hz) which provides a rich and objective data resource to assess movement patterns . Both vertical axis and triaxial counts from activity accelerometers can provide biologically meaningful data for assessing movement intensity, and hence energy expenditure [12, 13]. Calibration methods based on energy expenditure at different acceleration counts are then used to classify the movement as physically active versus sedentary. However, the ActiGraph does not provide information on body posture. On the other hand, the thigh-worn activPAL (PAL Technologies, Glasgow, UK) is a frequently used accelerometer to measure body posture [14, 15], classifying behavior as sitting (i.e. all non-upright postures), standing and stepping. Thus, measurements from either of these devices alone, often, do not provide consistent information about SB, and could result in a loss of information regarding SB patterns.

Exploiting the variety of data available in SB sensors, the goal of our current study is to implement Functional Principal Components Analysis (FPCA) to quantify the pattern and variation of movement (by ActiGraph accelerations) during activPAL-identified sitting events. Our unique approach uses the time-matched Actigraph and activPAL continuous datastreams, to extract sitting posture events and then applies FPCA to minute-level triaxial activity counts within sitting time. As a comparison, we also calculated the Posture and Physical Activity Index (POPAI) [6], which was proposed to classify each minute of activPAL sitting or standing as *inactive* or *active* by using a cut-point of vertical axis (VA) activity counts from ActiGraph. A salient advantage of FPCA, as we will demonstrate in this work, is that it utilizes the entire time series data, does not require pre-defined cut-points, captures the principal directions of variation, and achieves dimension reduction [16, 17]. While FPCA methods have been used successfully in physical activity research [17–19], to our knowledge they have not been extensively applied in SB research, especially in the context of jointly examining movement and posture.

As a proof of concept of the potential applications of FPCA to reveal novel insights between SB and health, we examine associations between FPCA-derived patterns and health-related outcomes, such as blood pressure. Studies have shown a positive relationship between prolonged sitting and blood pressure [20–23]; however, less is known about patterns of movement during sitting, and if/how these might impact health. As a complementary analysis, we also applied POPAI and compared health-related associations between methods.

## Method

### Study sample

Rise for Health (ClinicalTrials.gov: NCT03473145) [24] was one of the projects within the National Institute of Aging Program Grant “Sedentary Time and Aging Research (STAR)” at University of California San Diego aiming to provide more rigorous and comprehensive evidence on how to interrupt sitting time to improve health among overweight postmenopausal women. Overweight older women, spend the majority of their waking hours sitting, which increases their risk of chronic diseases. Engaging in moderate-to-vigorous physical activity can be challenging for this group. Therefore, the Rise for Health study was designed to understand the health benefits of decreasing sedentary behavior in this group. The primary aim of this 3-arm randomized controlled trial was to investigate how 3-month changes in sitting time or changes in brief sit-to-stand transitions would impact biomarkers of healthy aging, and physical, emotional, and cognitive functioning compared to an attention control condition.

For the current analyses, we used baseline (pre-randomization) data from 327 (recruited during 2018-2021) Rise for Health participants. Details on eligibility and study protocols have been previously published [24]. Pertinent to the current analysis, Rise for Health participants were instructed to wear an activPAL (at thigh) and ActiGraph GT3X+ (at waist) simultaneously for 24 hours a day for 7 days under free-living conditions and to track their daily sleeping time on a paper log.

### Device data processing

Event files from the activPAL were extracted using the VANE classification algorithm (PALanalysis, v8), which uses the thigh location of the device to identify sitting, standing, and stepping events. Daily waking wear time was identified as the complement of the sleeping time from the paper log. Waking wear sitting bouts (i.e., intervals with uninterrupted sitting) were determined by matching the timestamp of sitting activity from activPAL event file with the participant’s daily waking wear time.

The 1-second count file of ActiGraph GT3X+ with low-frequency-extension (LFE) filtering was generated with ActiLife software [25]. LFE option increases sensitivity to very low amplitude activities, such as slow walking, which might occur in elderly populations and our study sample [6, 26]. Non-wear time from the 1-second count file could be identified by the Choi algorithm [27] based on consecutive zero counts. Although most of the non-wear time detected by the Choi algorithm would overlap with participants’ sleeping time, the Choi algorithm could capture additional non-wear time during a participant’s waking wear time. Hence, the concurrent waking wear sitting bouts started with time-matching event file (activPAL) and count file (ActiGraph) and excluded not only the sleeping-time based on the sleeping log, but also any additional non-wear time detected by the Choi algorithm. The 1-second-level activity counts from vertical, horizontal, and perpendicular axes (VA, HA and PPA) were then summed up over each minute, respectively; the minute-level triaxial vector magnitude (VM) was computed as $\sqrt{\textrm{VA}^2+\textrm{HA}^2+\textrm{PPA}^2}$ [25, 28]. For bout lengths that were not a multiple of a minute, the following rule was applied: if the final fraction of the bout was less than 30 seconds, that final fraction was removed; otherwise, say 40 seconds was the last fraction, activity counts were calculated at 40-second level and then multiplied by 60/40 to approximate the last minute counts of the sitting bout. Thirteen out of 327 participants had no concurrent waking wear data. To simplify the description, the term “concurrent waking wear sitting bouts” and “concurrent waking wear sitting time” were abbreviated as “sitting bouts” and “sitting time”, respectively, throughout the rest of the paper, unless otherwise specified.

Previous population-based studies have suggested that daily waking sitting time in adults typically ranged between 5 and 8 hours with self-reported measures and were higher with device measures, 7.7 to 11.5 hours [29]. Among those age > 80 years, the daily sitting time could be more than 13 - 14 hours [30, 31]. Hence, valid days were defined as concurrent waking wearing days of both devices, and days with total daily sitting time between 5 to 15 hours; about 5% of the participant-days were excluded. The minimum required number of valid day(s) per participant in the analyses was one. Furthermore, only sitting bouts $\le$ 1 hour within valid days, defined as valid sitting bouts, were utilized for analyses; 5% of the sitting bouts were then eliminated. The first reason that 1 hour was chosen as the upper limit was because the 95th percentile of the sitting bouts was 61 minutes. The second reason was for model fit feasibility which is described in more detail in the statistical analysis “Multilevel functional principal component analysis (MFPCA)” section. The final sample size was 314 and final valid participant-days was 1776. On these valid participant-days, the daily standing and stepping time were obtained based on the activPAL event status as well as participants’ daily waking wear time. The average daily non-sitting time was the mean of the total daily standing and stepping time over the valid wearing days per participant.

### Posture and Physical Activity Index (POPAI)

The POPAI-based inactive and active sitting time were calculated per sitting bout first, i.e., for each minute within a sitting bout if VA counts < 75 cpm (counts per minute), the minute was classified as inactive sitting, and as active sitting otherwise [6]. Average daily inactive and active sitting time were calculated as each participant’s sum of inactive sitting time or active sitting time, respectively, across all valid sitting bouts, divided by their total number of valid wear days.

### Health outcome and baseline characteristics

Blood pressure (BP), including systolic and diastolic (SBP and DBP), were the health outcomes of interest in the current study. They were measured at least 3 times at the participant’s pre-randomization clinic visit by trained study staff using a digital BP monitor (such as Dinamap or Accutor 7 or Dinamap V100). Participants were seated and at rest for at least 5 minutes prior to testing. Participants had their feet planted on the floor and arm on a table with their palm up during each of the blood pressure tests. The readings were taken at intervals of at least 1 minute apart. A fourth measurement was taken if two of three readings were more than 5 mmHg apart for each SBP and DBP. The mean value of all available measures was taken for SBP and DBP, respectively. Other baseline characteristics included in the analyses were age, race (white versus non-white), education (college and above versus below), employment status (work versus not working), body mass index (BMI) and hypertension status (yes/no). These characteristics were self-reported, except for BMI (weight and height were measured by study staff).

### Statistical analysis

#### Multilevel functional principal component analysis (MFPCA)

Our accelerometer-based activity data was not only high dimensional and irregularly-spaced, but also measured on multiple days per participant at baseline, which demanded a multilevel model to differentiate variations due to the hierarchical structure: participant-specific variation (level 1, between-subject) and day-specific variation (level 2, within-subject).

The time of day when a sitting bout of a particular length occurs will vary across participants, making it difficult to compare patterns if bouts are based on clock-time. Hence, we first ordered sitting bouts per participant per day by unique length from 1 minute to 60 minutes with an increment of 1 minute. We then implemented MFPCA to VM counts/minute within (ordered) sitting bouts; if a participant had multiple bouts with the same length in a day, the VM counts/minute were averaged, and if a participant did not have a certain bout length in a day, the empty bout was retained with each minute of VM counts marked as “missing”. This configuration placed all participants and all days under the same scale in terms of sitting bouts and their VM counts, and thus allowed us to compare counts within bouts of identical length across days and participants.

**Fig. 1:**
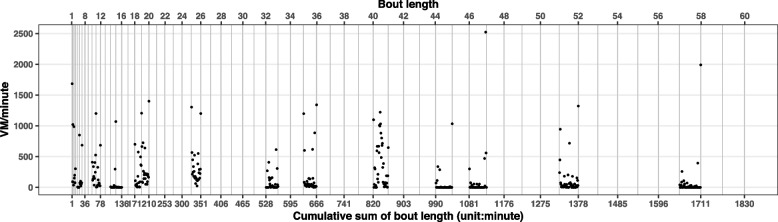
Example of one participant’s profile for one day of VM counts/minute within ordered sitting bouts. Vertical grey lines indicated all possible sitting bout lengths from 1 minute to 60 minutes. The corresponding horizontal axis at bottom was the cumulative sum of the top bout lengths and the maximum value of the bottom axis was $\sum _{b=1}^{60}b=1830$ minutes, which implied $t\in [1,1830]$ with increment of 1 minute. t = 1 at the bottom was mapping to 1-minute sitting bout at the top; t = 2 and 3 at bottom were mapping to the first minute and second minute of the 2-minute sitting bout at the top; t = 4 to 6 at the bottom would be the 3-minute bout at the top, etc. If the top bout length was 8 minutes, the corresponding bottom values would span from $\sum _{b=1}^7 b = 28$ to $\sum _{b=1}^8 b = 36$; if the top bout length was 60 minutes, the bottom values would span from $\sum _{b=1}^{59} b = 1770$ to 1830. Each minute of the bottom axis held a VM count value or a missing value. Black dots represent VM counts/minute within sitting bouts

Figure 1 illustrated an example of one participant’s profile for one day of VM counts/minute within ordered sitting bouts. Vertical grey lines indicated sitting bouts from length 1 minute to 60 minutes labeled by the top horizontal axis. As the bout length increases, so does the gap between the lines. This axis was important as it corresponds to ordered sitting bout lengths, and thus could be meaningfully interpreted. The corresponding horizontal axis at the bottom was the cumulative sum of the top bout lengths, and was necessary for the mathematical formulation of the FPCA model. Each unit of the bottom axis was aligned with a value of VM counts/minute or a missing value, which served as continuous variable *t* in the MFPCA formulation (see Eq. 1 for details). Black dots represent VM counts/minute within sitting bouts. Empty bouts, bouts without VM counts, for this participant on this day could be non-empty and have VM counts for other participants and/or on other days. This was another reason that maximum sitting bout length was set at 60 minutes, namely, to prevent excessive numbers of empty sitting bouts at the right tail.

To account for the hierarchical nature of these data (multiple days per participant), we adopted the MFPCA approach [16], which was designed to decompose the total variation into between- and within-subject levels and extract major modes of variation at both levels. The full model of MFPCA [16] was formulated as

1 $$\begin{aligned} X_{ij}(t) ={} & {} \mu (t) + \eta _j(t) + \sum \limits _{k=1}^{N_1}\xi _{ik}\phi _k^{(1)}(t) \nonumber \\{} & {} + \sum \limits _{l=1}^{N_2}\zeta _{ijl}\phi _l^{(2)}(t) +\epsilon _{ij}(t) \end{aligned}$$

where $X_{ij}(t)$ was the VM counts function measured over *t* (the bottom indexes in Fig. 1) for day *j* within participant *i* ($i=1,2,...,n, j=1,2,...,d_i$ where *n* was the total number of participants, $d_i$ indicated number of valid days for participant *i*); $\mu (t)$ was the overall mean count function; $\eta _j (t)$ was a day-specific shift from overall mean, and $\epsilon _{ij}(t)$ was an error term assumed to have a normal distribution $N(0,\sigma ^2)$. $\phi _k^{(1)}(t)$ and $\phi _l^{(2)}(t)$ were the eigenfunctions at level 1 ($k^{th}$ component) and level 2 ($l^{th}$ component); $\xi _{ik}$ and $\zeta _{ijl}$ were principal component (PC) scores at level 1 and 2, assumed to have normal distributions

$$\begin{aligned} \xi _{ik} \sim N(0,\lambda _k^{(1)}), \zeta _{ijl} \sim N\left( 0,\lambda _l^{(2)}\right) \end{aligned}$$

where $\lambda _k^{(1)}$ and $\lambda _l^{(2)}$ were the eigenvalues at level 1 ($k^{th}$ component) and 2 ($l^{th}$ component). Intuitively, each eigenfunction could be conceptualized as identifying a specific pattern of activity counts (over sitting bouts), while each subject’s corresponding PC score indicates to what extent that subject subscribes to this pattern with the eigenvalue quantifying the variance of the PC score. The PC scores were essential quantities in our analyses because they captured the signals of sitting patterns. $N_1$ and $N_2$ were the number of components retained at level 1 and level 2. The choice of $N_1$ and $N_2$ is usually based on a balanced selection between ensuring that enough variation in the data is explained, while also avoiding noise. This is a trade-off between under-fitting and over-fitting: retaining sufficient amount of the information from the data while reducing the chance of identifying spurious patterns. In our application, we required that the total variation explained by the number of components was 90% as in [16]. MFPCA was implemented using R package refund (version 0.1-26) [32].

#### Multiple linear regression (MLR)

We fit multiple linear regression models to investigate associations between movement patterns during sitting (i.e., the PC scores from the MFPCA) and blood pressure. Since blood pressure was not measured on multiple days during baseline, only the participant-level (level 1) PC scores were applied to the regression modeling focusing on subject-level effects on the outcome [17, 33]. The association between PC scores and blood pressure were examined in MLR controlling for participants’ baseline characteristics, as well as, two additional covariates: number of days with valid concurrent device wear and average daily non-sitting time.

Similarly, the association between POPAI-based sitting time (classified as active: vs inactive) with blood pressure were also assessed in MLR replacing PC scores with average daily inactive sitting time and average daily active sitting time in the model.

To better understand and compare the magnitude of the associations, PC scores and POPAI-based variables were standardized, and the MLRs were refit, respectively. Although the re-estimated coefficients would be different for these standardized variables, *p* values will be unaffected.

The overall $\alpha$ level of multiple comparisons at 0.05 was controlled by Benjamini and Hochberg false discovery rate (BH FDR) [34]. Both original *p* values and BH FDR adjusted *p* values (p-FDR) were presented. Implementing such correction improved the rigor of the approach. Model fit of MLRs were evaluated via residual and leverage plots.

#### Assessing goodness of fit

To provide an empirical evaluation of the MFPCA model, we superimposed and graphed observed data and fitted curves. The observed VM counts/minute within sitting bouts were plotted on a participant-day, then we added one element at a time for fitted curves based on model Eq. 1 as follows: 1. the overall mean, $\mu (t)$; 2. mean day shift, $\mu (t)+ \eta _j(t)$; 3. the participant level fitted curve, $\mu (t)+ \eta _j(t)+ \sum _{k=1}^{N_1}\xi _{ik}\phi _k^{(1)}(t)$; 4. the participant-day level fitted curve, $\mu (t)+ \eta _j(t)+ \sum _{k=1}^{N_1}\xi _{ik}\phi _k^{(1)}(t)+ \sum _{l=1}^{N_2}\zeta _{ijl}\phi _l^{(2)}(t)$.

#### Assessing impact of missing data in MFPCA

We used a (pseudo-) simulation approach to evaluate the robustness of our approach to missing data, i.e., absence of a sitting bout of a particular length, which was relatively common in our dataset, especially for longer bouts. To describe the extent of missingness, we introduce some notation. Let $b = 1,2,\dots ,60$ denote the index (and the length) of the sitting bouts and $m_b$ the number of participant-days that did not register a *b*-minute bout. For instance, $m_1$ = 14 and $m_{60}$ = 1655 in our data implied unregistered rate (with denominator 1776) were 0.8% and 93%, respectively; the average unregistered rate across all 60 indexes was 71%. The overall missing rate of VM counts/minute from the 1776 $\times$ 1830 data matrix was $\frac{\sum _{b=1}^{60}(m_b \, \times \, b)}{1776\times 1830}= 83\%$, i.e. there were a considerable number of missing data across participant-days. Although MFPCA can accommodate data missing at random, simulations were carried out to evaluate the impact of large amount of missingness in constructing PCs, especially PC1 at level 1 since this component accounted for a majority of the variability at subject level. Details were described in the Supplementary materials (A.2).

All analyses were performed in the R statistical programming language (4.2.3) [35].

## Results

### Sample characteristics

**Fig. 2:**
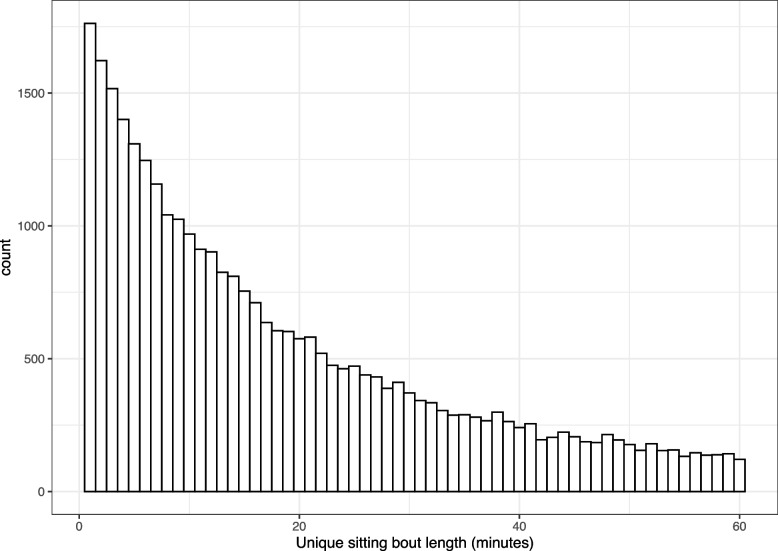
Histogram of sitting bouts from 1 minute to 60 minutes among all participant-days (1776)

The total number of participants included in the analyses was 314; the number of days with valid concurrent wear of both devices for each subject varied from 1 day to 9 days with average 5.7 days (median: 6 days); number of weekdays per participant ranged from 1 to 7 with average 3.8 (median: 4); number of weekend days ranged from 1 to 4 with average 1.9 (median: 2). The total number of participant-days was 1776, 579 were from weekends and 1197 were from weekdays, giving a 2.4:5 ratio of weekend:weekdays. Figure 2 showed the histogram of sitting bouts from 1 minute to 60 minutes among all participant-days. For example, as mentioned in “Assessing impact of missing data in MFPCA” section, among 1776 participant-days, 1762 had 1-minute sitting bout (the remaining 14 participant-days did not register 1 minute bouts), and 121 had 60-minute sitting bout (the remaining 1655 participant-days did not). The 2.5 percentile and 97.5 percentile of the sitting VM/minute were 0 and 1021, with median 20 and mean 141 among all participant-days.

The descriptive statistics of blood pressure, daily activity time and other baseline characteristics are presented in Table 1. The average age of the participants was 68 years and their average BMI was 32.3. The mean SBP and DBP were 127.2 mmHg and 75.6 mmHg, respectively. The vast majority of participants were white (92.2%) and slightly over half of the participants (52.3%) reported having a hypertension diagnosis at baseline. Average daily inactive sitting and active sitting time based on POPAI were 307.7 minutes and 94.6 minutes, respectively; hence, 23.4% of the sitting time was active sitting in this sample. Average daily non-sitting time (including standing and stepping time) was 262.6 minutes.

**Table 1:** Descriptive statistics of sample characteristics

	Mean (SD^a^)		Count (%^c^)
SBP (mmHg)	127.2 (16.0)	Race (white)^b^	284 (92.2)
DBP (mmHg)	75.6 (10.5)	Education (college and above)	222 (72.1)
Age (years)	68.2 (7.3)	Employment (working)	123 (39.9)
BMI	32.3 (4.9)	Hypertension (yes)	159 (52.3)
Average daily time (minute)			
inactive sitting	307.7 (78.7)		
active sitting	94.6 (40.6)		
non-sitting	262.6 (91.0)		
standing	193.7 (73.8)		
stepping	68.9 (27.1)		

^a^Standard deviation (SD)

^b^Binary variables: Race (white versus non-white), Education (college and above versus below), Employment status (work versus not working), Hypertension status (yes/no)

^c^Among 314 participants, % was based on the non-missing corresponding variables at baseline, hypertension status had 10 missing values while all others had 6

### Multilevel functional principal components

Two components at participant-level (level 1, $N_1$=2) and six components at day-level (level 2, $N_2$=6) explained at least 90% of the total variance, in which 29% was attributed to level 1 and 71% was to level 2.

#### Participant-level (level 1) principal components

**Fig. 3:**
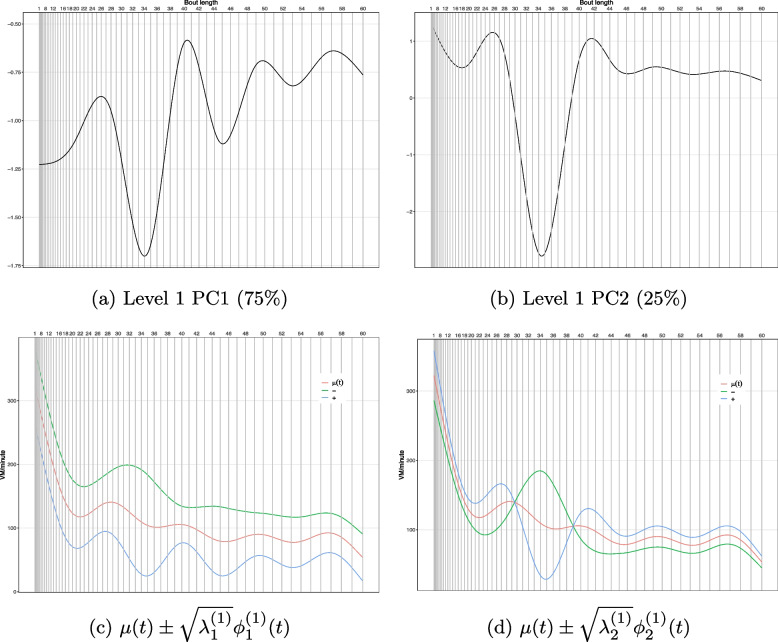
Participant-level eigenfunctions (top) and mean function $\mu (t)$ (red) with addition (blue) or subtraction (green) of square root of two eigenvalues multiplying corresponding eigenfunctions (bottom)

Figure 3a and b showed the 2 PC eigenfunctions at the participant-level, $\phi _1^{(1)}$ and $\phi _2^{(1)}$, as well as the proportions of the variability accounted by the 2 PCs at this level (75% and 25%). $\phi _1^{(1)}$ was negative across the entire x-axis. This indicated that participants with larger PC scores tended to have lower VM counts/minute than the overall mean, hence, less acceleration or movement. $\phi _2^{(1)}$ showed both positive and negative values: positive when bout length was short (less than 30 minutes) and became negative at medium bout length (around 30 - 39 minutes), then positive at longer bouts (> 39 minutes - 60 minutes). Interestingly, the most negative valley of $\phi _1^{(1)}$ and $\phi _2^{(1)}$ curves appeared at the same area, around sitting bout length 34 minutes, suggesting the sitting bout at that length could be important for capturing between-subject variability. Figure 3c and d illustrated the overall mean function, $\mu (t)$ (red), with addition (blue) or subtraction (green) of square root of eigenvalues multiplying corresponding eigenfunctions, e.g., $\mu (t) \pm \sqrt{\lambda _1^{(1)}}\phi _1^{(1)}(t)$ (left) and $\mu (t) \pm \sqrt{\lambda _2^{(1)}}\phi _2^{(1)}(t)$ (right), which could be interpreted as the weighted deviation away from the overall mean captured by the 2 PCs. The predicted mean function curve of VM counts decreased rapidly during short bouts and then stayed relatively stable afterwards. Aligned with Fig. 3a and b both components showed the largest variance of VM counts at a sitting bout length of 34 minutes.

#### Day-level (level 2) principal components

The 6 day-level (level 2) PCs represented random day-level functional shift from the participant level curve. The eigenfunctions exhibited oscillatory pattern and captured day to day variation in movement during sitting bouts. A detailed description and figures are shown in the Supplementary materials (A.1).

#### Model fit of MFPCA

**Fig. 4:**
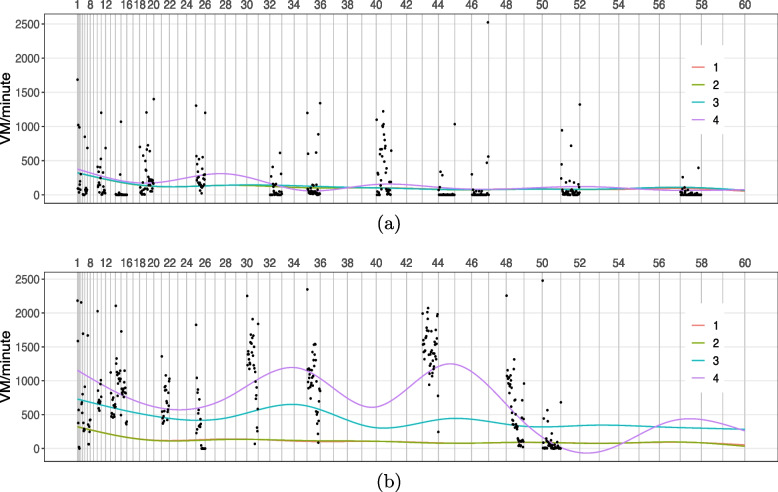
Example fitting profiles in different participants on different days of MFPCA curves tracing the observed VM counts/minute (black dots) within sitting bouts by adding one element at a time based on model Eq. 1: 1. $\mu (t)$; 2. $\mu (t)+ \eta _j(t)$; 3. $\mu (t)+ \eta _j(t)+ \sum _{k=1}^{2}\xi _{ik}\phi _k^{(1)}(t)$; 4. $\mu (t)+ \eta _j(t)+ \sum _{k=1}^{2}\xi _{ik}\phi _k^{(1)}(t)+ \sum _{l=1}^{6}\zeta _{ijl}\phi _l^{(2)}(t)$. **a** was the same participant-day from Fig. 1 where a majority of VM counts were below 1000 cpm with notable size of those close to 0; **b** was a different participant on a different day where a large number of VM counts were still above 1000 cpm and fewer VM counts were close to 0

Figure 4 showed illustrative examples of two participant-days: (a) was the same participant-day from Fig. 1 where a majority of activity counts were below 1000 cpm with a large number of counts around 0; (b) was a different participant on a different day where a large number of activity counts were above 1000 cpm and fewer were around 0. The figures showed the contributions of principal components from both levels in terms of capturing the activity patterns, and demonstrated that the fitted curve incorporating both participant and day-level components tracks the observed data well.

As for impact of missing data in MFPCA, comparison from simulated complete data and incomplete data suggested that level 1 PC1 from incomplete data still captured sufficient variability at subject level despite the large amount of missingness. More details were shown in the Supplementary materials (A.2) and Table A1.

### Regression association

Two sets of MLR were conducted; while the focus was on the first set assessing the associations between level 1 principal component scores (PC1, PC2) and blood pressure, the second set examined the associations of POPAI based metrics – average daily inactive sitting time and active sitting time – with blood pressures (Pearson correlation between average daily inactive sitting time and active sitting time was 0.01). Both sets of models were adjusting for covariates: age, race (ref: non-white), education (ref: below college), employment status (ref: not working), BMI, reported hypertension status (ref: no), as well as number of concurrent wear days of devices and average daily non-sitting time (Table 2). Diagnostic plots displayed neither notable violations of modeling assumptions nor influential outliers (plots not shown), indicating that the fit of the MLR models was adequate.

In general, DPB displayed more compelling evidence of associations with both sets (MFPCA based and POPAI based) of sitting pattern variables than SBP did. The strongest association was shown between DBP and level 1 PC1, the higher the PC1 values, the higher the DBP (p = 0.0009, p-FDR = 0.007), indicating a positive relationship. Recall that the level 1 PC1 eigenfunction was negative across the entire domain, which implied that participants with higher PC1 scores tended to have lower VM counts (and thus are more inactive). Hence, our regression results suggest that lower VM counts were associated with worse DBP values. Standardizing both PC scores and refitting the MLR revealed the re-estimated $\hat{\beta }$ (s.e) of PC1 score was 2.041 (0.607) suggesting 1 unit increase in level 1 PC1 was associated with 2.04 mmHg increase in DBP. No signficiant associations were detected between PC scores and SBP.

Similar positive association was also observed between DBP and POPAI average daily inactive sitting time (p = 0.008, p-FDR = 0.03). Each minute increase in daily inactive sitting time was associated with 0.02 mmHg increase in DBP (in other words, each hour increase in daily inactive sitting time was associated with a 1.20 mmHg increase in DBP). Standardizing both inactive and active sitting time, the re-estimated $\hat{\beta }$ (s.e) of inactive sitting time was 1.636 (0.614) indicating 1 unit increase in daily inactive sitting time was associated with a 1.64 mmHg increase in DBP. In addition, there was a trend between SBP and POPAI average daily inactive sitting time, where each hour increase in daily inactive sitting time was associated with 1.50 mmHg increase in SBP (*p* = 0.03, p-FDR = 0.08).

**Table 2:** Association of blood pressure with MFPCA/POPAI variables from MLR

Outcome	Estimate	MFPCA		POPAI	
		Level 1 PC1	Level 1 PC2	Average daily inactive sitting time	Average daily active sitting time
SBP	$\hat{\beta }$ (s.e)^1^	0.014 (0.017)	-0.026 (0.027)	0.025 (0.011)	-0.018 (0.021)
	p^2^	0.42	0.34	0.03	0.40
	p-FDR^3^	0.42	0.42	0.08	0.42
DBP	$\hat{\beta }$ (s.e)	0.040 (0.012)^4^	0.034 (0.019)	0.021 (0.008)^5^	-0.030 (0.015)
	p	0.0009	0.07	0.008	0.05
	p-FDR	**0.007**	0.11	**0.03**	0.10

^1^ Parameter estimates and their standard errors (s.e) from the MLR between independent variables (MFPCA or POPAI variables) and outcomes (SBP or DBP) controlling for other baseline characteristics, including age, race (ref: non-white), education (ref: below college), employment status (ref: not working), BMI, hypertension status (ref: no), as well as concurrent wearing days of devices and average daily non-sitting time

^2^ p : *p* values from MLRs

^3^ p-FDR : adjusted *p* values based on Benjamini and Hochberg false discovery rate (BH FDR) [34] accounted for 8 pairwise comparisons

^4^ Standardized $\hat{\beta }$ (s.e) = 2.041 (0.607) between DBP and level 1 PC1

^5^ Standardized $\hat{\beta }$ (s.e) = 1.636 (0.614) between DBP and inactive sitting time

## Discussion

The current study brought the unique design and application of FPCA into the field of accelerometer data in SB research. While most research has implemented FPCA on physical activity accelerometer (ActiGraph) data to explore temporal or intensity activity patterns, we time-matched both posture accelerometer and physical activity accelerometer data to scrutinize movement within the core event of SB, sitting. The novel construction of activity counts within posture-based sitting bouts placed all subjects and all wearing days under the same scale, hence, PC scores of each component are able to capture variation in movement within sitting bouts and thus are more interpretable. To demonstrate potential applications of this FPCA approach in public health, the current study assessed the person level PC scores and revealed the evidence that sitting with less movement was associated with higher DBP. While the POPAI based analyses also showed positive association between inactive sitting time and higher DBP, it requires selection of a specific cut-point threshold (75 cpm in this case) to delineate active vs inactive sitting. Although use of cut-points are very common in SB research, there is lack of agreement and consensus on the “best” cpm cut-points [36–38]. For instance, while a widely applied cut-point for adults wearing ActiGraph GT3X is VA < 100 cpm as SB [38], Aguilar-Far$\acute{i}$as et al. [39] suggested VA < 25 cpm and Kozey-Keadle et al. [14] suggested VA < 150 cpm as SB. The difference can be substantial. Another limitation of cut-point methods is that dichotomization does not make full use of the data in the accelerometer signal beyond or below the cut-point. These drawbacks can potentially attenuate or exaggerate the relationships of SB with health outcomes [40]. On the other hand, FPCA transforms the original functional data to a set of asymptotically equivalent independent PC scores and yields a parsimonious representation of the original data [41, 42]. The stronger signal in Table 2 for the level 1 PC1 could be an indication that FPCA grasped more information than the method based on the cut-point. Besides dimension reduction, FPCA attempts to characterize the dominant modes of variation of random trajectories around their overall mean [42] (demonstrated in Fig. 3c, d and Supplementary Fig. A1b). Hence, it provides a robust alternative to study SB.

Given the increasing use of machine learning (ML) techniques in health behavior research, it is important to clarify the unique contributions of FPCA in this context. To our knowledge, current applications of ML (including our own work) [43–45] focus largely on posture or activity detection (e.g., siting, standing, walking etc), and hence involve binary or categorical classification of behaviors. FPCA on the other hand, uses the continuous data stream from the device to identify the main sources of variation in (movement) patterns, and is agnostic to categories of behavior. As such, MFPCA and ML provide complementary advantages: ML can identify the behavior, and FPCA can elicit variation in movement during the behavior. Both methods are useful for understanding and quantifying human movement and associated health outcomes.

There are several limitations in our current study. First, FPCA is an exploratory statistical approach, and in this study, we investigated cross-sectional FPCA-based sitting patterns in relation to the health outcomes at baseline. Rise for Health [24] is a longitudinal randomized controlled trial, hence, the natural next step would be to extend the MFPCA model implemented in the current work to a 3-level model, in which participant is the level 1, visit (baseline and final visit) is the level 2 and day is the level 3. The extension will not only bring us richer functional data to model dynamic changes in movement patterns, but will also allow us to compare outcomes across different arms, control (healthy aging) and two intervention groups: reducing sitting and increasing sit-to-stand transitions. Besides blood pressure, additional health-related outcomes can be considered. However, there are methodological complexities in extending the current MFPCA model to a longitudinal setup; we aim to explore this extension in future work. Second, in the current study, the day level PC scores were not incorporated into the association assessment, mainly because clinical data (outcomes) were not collected at the day-level. Applications that incorporate the day-level PCs could offer further insights on sitting patterns. Third, the current daily sleeping time was self-reported, which could be susceptible to recall, response and social desirability bias [38]. In the recent release of activPAL scoring software, the built-in algorithm can now identify “time in bed” start and “time in bed” end, which would facilitate our proposal to automate sleep time identification and removal in a more consistent fashion in the future. Fourth, our samples were overweight sedentary postmenopausal women, and majority were white and highly educated (i.e., college and above), which might not represent a broader older adult population. Lastly, the current MFPCA model was built based on minute level VM counts to align with the POPAI-based approach [6]. In theory, it is possible to extend the model to different epoch lengths, such as 15 seconds. However, the computational feasibility might become a major challenge.

To illustrate the potential application of our approach to public health studies, we evaluated associations between the FPCA-derived SB patterns and blood pressure, and found that less movement during sitting was associated with higher diastolic blood pressure. While our health-related analysis was largely a proof of concept, our results have face validity. Prolonged sitting time, has been linked to increased blood pressure, especially after the age of 45 years in men and 5-10 years later in women, often after menopause [21, 23, 46, 47]. Reducing or interrupting prolonged sitting time (either by walking or taking standing breaks) has been shown to have SBP or DBP-lowering effects [22, 48–51]. Previous studies in office environments have recommended workplace interventions to break up prolonged SB by dynamic chairs to encourage movements [52, 53]. It has been shown that energy expenditure increased significantly either using an under-table leg-fidget bar or a fidget-promoting chair compared to the standard office chair [54]; hence, one of the approaches could be to render sitting more active, called “dynamic sitting”, to provide an alternative for when standing or getting up from a desk is not feasible [53, 54]. While the types of active sitting in these previous studies may differ from our study, our findings support a proposition of replacing more inactive sitting with active sitting. We emphasize that we are not recommending replacing physical activity with active sitting. However for some populations, such as highly sedentary, elderly, and/or overweight individuals as in our study sample, or those with comorbidities or physically disabled subpopulations, adhering to physical activity guidelines may not be feasible. For these populations light activity and active sitting may provide viable alternatives and a more achievable path to healthy living. Studies have suggested that non-exercise activity thermogenesis (NEAT) has the potential to prompt energy expenditure over time with a higher rate of adherence [55]. More research, including longitudinal studies as well as intervention trials, are needed to further examine and evaluate the impact of active sitting patterns on health-related outcomes.

## Conclusion

To our knowledge, this is the first study to develop a MFPCA approach for examining movement during SB. The unique design of time-matching both the posture-based activPAL and movement-based ActiGraph accelerometer data and applying FPCA to triaxial activity counts within sitting time take advantage of the rich minute-level data rather than daily or weekly summary metrics, and furthermore avoids the use of cut-point thresholds at the same time. We believe this approach offers a powerful statistical tool to elucidate variation in SB patterns and health [56–58].

### Supplementary Information

**Supplementary Material 1.**

## Data Availability

The data that support the findings of this article are available from the corresponding author upon reasonable request. The R code of applying MFPCA to a simulated data is available on Github (https://github.com/rongw16/sitPatternMFPCA)

## Abbreviations

SB: Sedentary behavior
FPCA: Functional principal component analysis
MFPC: Multilevel functional principal component analysis
MLR: Multiple linear regression
POPAI: Posture and physical activity index
PC: Principal component
BP: Blood Pressure
SBP: Systolic blood pressure
DBP: Diastolic blood pressure
BMI: Body mass index
VA: Vertical axis
HA: Horizontal axis
PPA: Perpendicular axis
VM: Vector magnitude
STAR: Sedentary time and aging research
LFE: Low frequency extension
cpm: Count per minute
SD: Standard deviation
s.e: Standard error
BH FDR: Benjamini and Hochberg false discovery rate
p-FDR: Benjamini and Hochberg false discovery rate adjusted *p* value
NEAT: Nonexercise activity thermogenesis
MBE: Mean bias error
RMSE: Root Mean squared error
NRMSE: Normalized root mean squared error
SEM: Standard error of mean
