# Supplementary material for: Using functional principal component analysis (FPCA) to quantify sitting patterns derived from wearable sensors
Source: Int J Behav Nutr Phys Act. 2024 Apr 26;21:48. doi: 10.1186/s12966-024-01585-8 (PMC11055353; doi:10.1186/s12966-024-01585-8)
Supplement: Supplementary file 1 — Supplementary Material 1. [file 12966_2024_1585_MOESM1_ESM.pdf]

Appendix A Supplementary materials

A.1 Level 2 Principal Components

Day-level (level 2) PCs revealed additional directions of variation. Figure A1a showed the six eigenfunctions at the day-level and the proportion of the variability accounted by each component at this level. These components represented the random day-level functional shift from the participant level curve. The eigenfunctions exhibited fluctuating patterns above and below zero, and captured day to day variation in movement during sitting bouts. Figure A1b showed the overall mean function, $\mu(t)$, with addition (blue) or subtraction (green) of square root of 6 eigenvalues multiplying corresponding eigenfunctions at this level.

A.2 Assessing the impact of missing data: a simulation study

Missing values were inevitable since fully non-missing data would require every subject to engage in all 60 unique sitting bouts (i.e., all lengths from 1 minute to 60 minute and thus register VM counts/minute in all 60 unique sitting bouts on all days), which was implausible. In order to assess the impact of missingness on our results, we used a pseudo-simulation approach. First we "created" a complete data set, and second removed data points to reflect the observed missingness patterns in our Rise for Health data application. We then implemented FPCA on this artificially created dataset with missing values, and compared the results to the complete dataset. The procedure is described in detail below.

To create a complete dataset, the following steps were carried out: (1) utilize the output of our MFPCA level 1 and level 2 principal components scores and eigenfunctions, as well as $\mu(t)$ and $\eta_j(t)$, to derive fitted $X_{ij}(t)$ based on model 1 in statistical analysis section; hence, each subject-day would have a fitted curve similar to the purple line in Figure 4; (2) for each fitted curve, a random error $\epsilon_{ij}(t)$ was added, where $\epsilon_{ij}(t) \sim N(0, \sigma^2)$ and estimated $\sigma^2 = 54457.75$ was from the output of MFPCA as well. This represented a 1776×1830 complete data matrix without missing values. Next, we identified the locations of missing values in our observed data as described in statistical analysis section and set those locations to missing in the complete data, which would create a 1776×1830 incomplete data matrix with the same proportion (83%) and same locations of the missingness as the observed data.

MFPCA was performed on the complete and incomplete data, respectively. As in the data application, the criteria for selecting the number of PCs was set as pve (proportion of variance explained) equal to 0.9. Pearson correlations and Mean Square Error (and related metrics detailed below) were used to compare the complete data and incomplete data MFPCA models.

Pearson correlation of level 1 PC1 scores from both models was 0.95 and correlation of PC2 was 0.69 (see Figure A2). Denoting ξ_{ik} as the ith subject's k^{th} PC score (k=1,2) at level 1 based on complete data (reference score) and $\hat{\xi}_{ik}$ as the corresponding subject's PC score based on incomplete data, define Mean Bias Error (MBE) as $\frac{\sum_{i=1}^{n}(\hat{\xi}_{ik} - \xi_{ik})}{n}$ where $n = 314$ and Root Mean Square Error (RMSE) as

$\sqrt{\frac{\sum_{i=1}^{n}(\hat{\xi}_{ik}-\xi_{ik})^2}{n}}$. Our simulations showed MBE was 0.96 for PC1 and -1.20 for PC2 at level 1; RMSE was 16.7 for PC1 and 26.0 for PC2. However, eigenvalues, which are the variances of the PC scores, from both models were large, for example, 2254 (complete data) and 2379 (incomplete data) for level 1 PC1. Normalized RMSE (NRMSE) has been proposed in previous studies on device data to evaluate the performance of different methods; RMSE was presented as a fraction of the overall range of the reference scores [1, 2]. Adapting the same quantity and utilizing the range of PC scores developed from complete data as normalizing denominator, our simulations showed NRMSE was 4.6% for PC1 and 7.0% for PC2. Thus the normalized error for PC1 is $< 5\%$, with slightly higher values for PC2, which is remarkably promising, given the large amount of missing data.

The computational time of MFPCA on complete data above was 68 days running on Nautilus [3] Kubernetes cluster requesting 64 GB RAM and 6 CPUs (it was less than a day for incomplete data). To further assess the variability of MBE, RMSE and NRMSE in level 1 PC1 between complete and incomplete data, we adopted a subset strategy (described below) instead of bootstrapping the entire sample, based on the computation feasibility of the former approach. A subset of 35 participants and 200 columns were randomly selected from complete and incomplete data, respectively; for each participant selected, all of her valid days were included as well. The ratio of 35:200 was roughly equal to those from the full dataset, 314:1830. The selection was repeated 10 different times, and each time the selected participants, participant-days and columns were identical in the complete and incomplete data. MFPCA was then performed on both subsets with pve = 0.9 as the criteria for determining the number of PCs selected. Of note, the random selection of columns for subsets would break the alignment and relationship between sitting bouts and VM counts/minute. However, the goal of the simulation was to assess the impact of missing VM counts/minute on construction of PCs, thus the structure of bouts was not essential here.

Summary Table (A1) and Figure (A3) shows comparisons of level 1 PC1 scores constructed from the 10 complete and incomplete subsets. Pearson correlations of PC1 scores ranged from 0.85 to 0.96 with average 0.92. Average MBE of PC1 was -0.21 with standard error of mean (SEM) 1.37, average RMSE was 18.6 with SEM 4.73 and average NRMSE was 8.3% with SEM 2.48%.

These simulations suggested that level 1 PC1 from incomplete data could captured sufficient variability at subject level even with large amount of missing data.

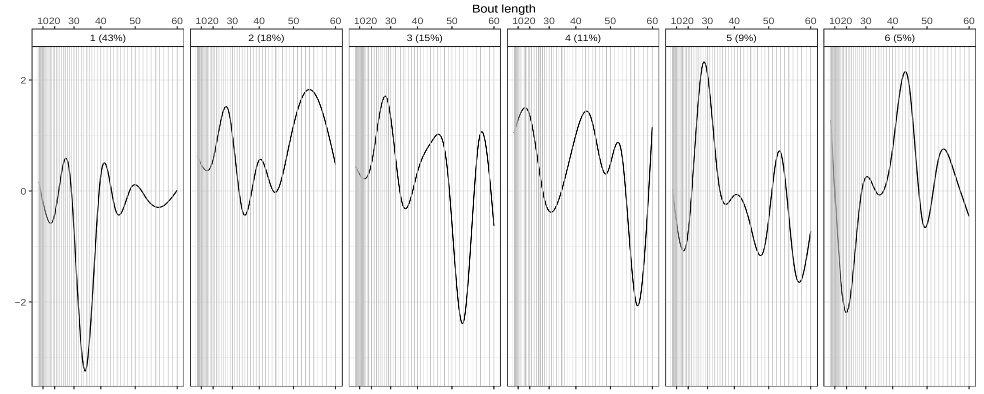

(a) Six day-level eigenfunctions and the variability explained by the components at this level

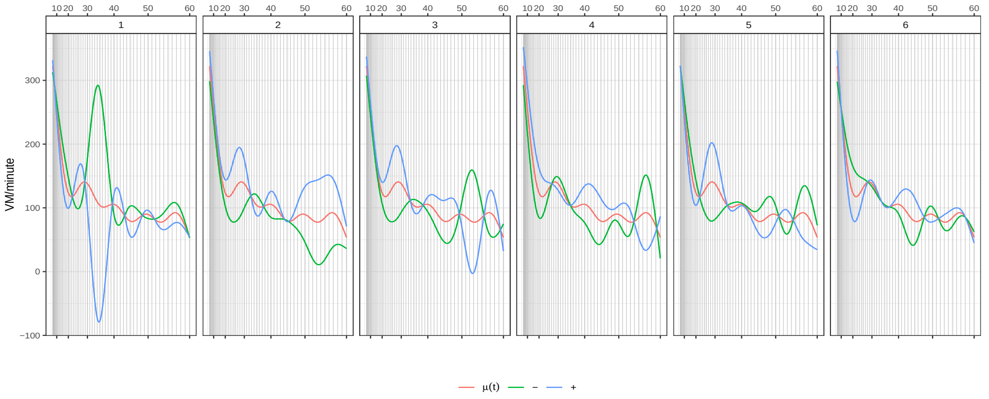

(b) Mean function, $\mu(t)$ (red), with addition (blue) or subtraction (green) of square root of eigenvalues multiplying corresponding eigenfunctions, e.g., $\mu(t) \pm \sqrt{\lambda_l^{(2)}}\phi_l^{(2)}(t)$, $l = 1,2,3,4,5,6$.

Fig. A1: Day-level (level 2) components

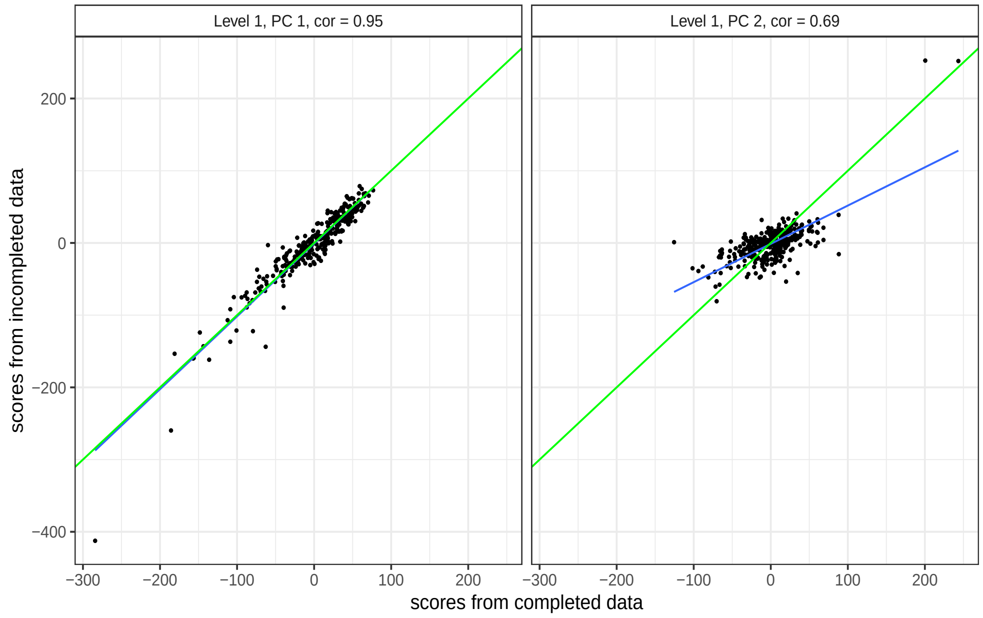

Fig. A2: Comparison of level 1 PC scores from complete data and incomplete data. Green line was diagonal line; blue line was regression line; cor: Pearson correlation.

Table A1: Simulation comparison of Level 1 PC1 from 10 complete and incomplete subsets

Simulation	Subset ratio[1]	VM missing rate (%)[2]	MBE[3]	RMSE[4]	NRMSE (%)[5]	Pearson correlation[6]
1	35:194:200	81.2	-0.70	31.00	9.1	0.91
2	35:206:200	82.7	-1.27	16.10	8.2	0.92
3	35:195:200	82.8	-0.42	16.34	5.8	0.95
4	35:200:200	81.0	0.50	19.06	8.3	0.90
5	35:195:200	82.7	-0.41	19.75	13.8	0.85
6	35:202:200	81.4	-2.19	15.88	7.0	0.95
7	35:203:200	82.3	-0.04	19.21	5.9	0.96
8	35:204:200	83.9	1.86	14.48	5.7	0.96
9	35:194:200	83.2	-0.64	15.40	9.7	0.90
10	35:198:200	83.3	1.22	18.85	9.7	0.90

[1] Ratio of participant : participant-days : columns in subsets; it was 314:1776:1830 in real data.

[2] VM counts/minute missing rate in incomplete subsets; it was 83% in real data.

[3] Mean Bias Error (MBE): $\frac{\sum_{i=1}^{35}(\hat{\xi}_{i1}-\xi_{i1})}{35}$, where $\hat{\xi}_{i1}$ was the ith subject's PC1 score at level 1 from incomplete subset and ξ_{i1} was the corresponding PC score from complete subset.

[4] Root Mean Square Error (RMSE): $\sqrt{\frac{\sum_{i=1}^{35}(\hat{\xi}_{i1}-\xi_{i1})^2}{35}}$.

[5] Normalized Root Mean Square Error (NRMSE): $100 \times \frac{\text{RMSE}}{\max(\boldsymbol{\xi}_{\cdot 1})-\min(\boldsymbol{\xi}_{\cdot 1})}$, where $\boldsymbol{\xi}_{\cdot 1}$ represented the vector of PC1 scores from complete subsets.

[6] Pearsonn correlation between level 1 PC1 scores from incomplete and complete subsets.

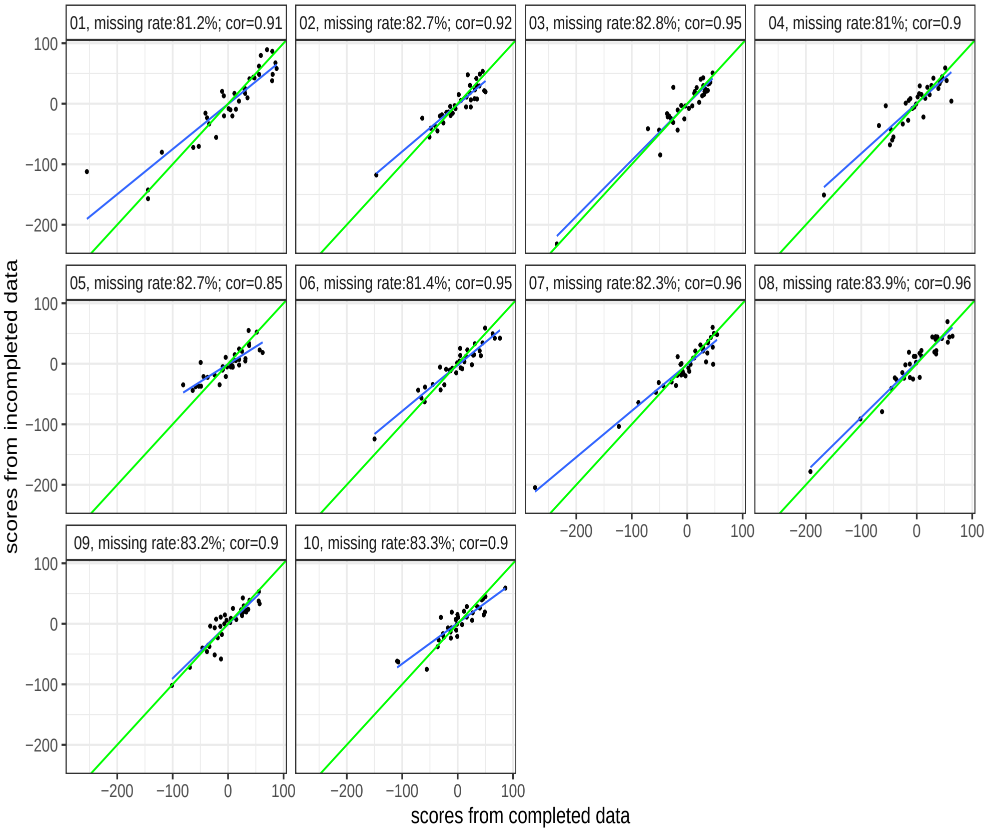

Fig. A3: Comparion of level 1 PC1 scores between 10 simulated complete and incomplete subsets. Green line was diagonal line; blue line was regression line; cor: Pearson correlation.

References

[1] Jacobs DA, Ferris DP. Estimation of ground reaction forces and ankle moment with multiple, low-cost sensors. Journal of neuroengineering and rehabilitation. 2015;12(1):1–12.

[2] Meinders E, Booij MJ, van den Noort JC, Harlaar J. How to compare knee kinetics at different walking speeds? Gait & Posture. 2021;88:225–230.

[3] National Research Platform (NRP), San Diego Supercomputer Center at University of California San Diego.: Nautilus. Accessed: 2023-01-30. https://portal.nrp-nautilus.io, https://www.sdsc.edu/services/hpc/nrp/index.html.
